# Network meta-analysis of adjuvant treatments for patients with hepatocellular carcinoma after curative resection

**DOI:** 10.1186/s12876-023-02955-5

**Published:** 2023-09-20

**Authors:** Yanyan Ye, Ying Wang, Haoqian Xu, Fengming Yi

**Affiliations:** 1 Department of Ultrasound, The Second Affiliated Hospital of Nanchang University, Nanchang, 330006 P.R. of China; 2 Department of Oncology, Second Affiliated Hospital of Nanchang University, Nanchang, 330006 P.R. of China; 3 JiangXi Key Laboratory of Clinical and Translational Cancer Research, Nanchang, 330006 P.R. of China

**Keywords:** Adjuvant treatment, Hepatocellular carcinoma, Recurrence, Survival, Network meta-analysis

## Abstract

**Purpose:**

The prevention of recurrence for patients with hepatocellular carcinoma after curative resection is still a great challenge in clinical practice. There are numerous studies that trying to search for favorable strategies to decrease the recurrence and prolong life span for these patients, whereas no consensus is reached till now. Herein, we aim to compare the efficacy between different reported treatments by network meta-analysis(NMA).

**Methods:**

We searched Pubmed, Web of Science and Cochrane Library for abstracts and full-text articles published from database inception through February 2023. All of the random controlled trials(RCTs) were evaluated and collected as eligible studies. The primary outcome was the prevention of recurrence between different procedures. The second outcomes were one-year survival, three-year survival and five-year survival.

**Results:**

Thirty-two RCTs including 5783 patients were selected, and 12 treatments were classified. Most of the studies were high quality with low bias. Thirty-one studies including 5629 patients were recruited for recurrence analysis. The network meta-analysis showed benefits from transarterial chemoembolization(TACE) + portal vein chemotherapy(PVC)[OR, 2.84 (1.15,6.99)] and internal radiotherapy(IRT) [OR, 2.63 (1.41,4.91)] compared to non-adjuvant(NA) treatment when considering prevention of recurrence. Seventeen studies including 2047 patients were collected for one-year survival analysis. The network meta-analysis showed benefit from TACE[OR, 0.33 (0.14,0.75)] when considering one-year survival. Twenty-one studies including 2463 patients were collected for three-year survival analysis. The network meta-analysis showed TACE [OR, 0.51 (0.30,0.86)], IRT[OR, 0.41 (0.20,0.83)] and dendritic cell(DC) [OR, 0.09 (0.01,0.98)] were better than NA when considering three-year survival. Sixteen studies including 1915 patients were collected for five-year survival analysis. The network meta-analysis didn’t show any benefit from different treatments when considering five-year survival. Other strategies including external radiotherapy(ERT), branched-chain amino acids(BCAA), hepatic artery infusion chemotherapy(HAIC), cytokine-induced killer(CIK), adoptive immunotherapy(AIT), Huaier, interferon(IFN), oral chemotherapy(OCT) and sorafenib(SOR) didn’t show significant benefit regardless of prevention of recurrence or short-, long- time survival.

**Conclusion:**

This NMA found that TACE + PVC and IRT were considered as the procedures to decrease HCC recurrence rate. TACE, IRT and DC were preferred when considering the extending of life span for post-operative patients with HCC. Large scale of RCTs are needed to verify it.

## Introduction

Resection is still one of the main strategies for the patients with early stage of hepatocellular carcinoma(HCC), and the expected survival of these patients could reach up to more than 5 years(Reig et al. 2022). However, nearly 50% of the patients will develop recurrent HCC within 5 year after surgical resection(Tabrizian et al. 2015; Yao et al. 2022), which decreases the overall survival(OS) in these patients and patients with unresectable or advanced stage indicate poor OS (Sun et al. 2019; Wang et al. 2019, 2022) A study based on large scale of investigation and follow-up demonstrated that preoperative alpha-fetoprotein(AFP) level higher than 400 ug/L, tumor size greater than 5 cm, multiple tumors, satellites, microvascular invasion, cirrhosis and intraoperative blood transfusion were considered as risk factors of tumor recurrences(Yao et al. 2022).

To decrease the recurrence rate, numerous strategies were applied to be adjuvant therapy for the patients after tumor resection, including adoptive immunotherapy(AIT), external radiotherapy(ERT), hepatic artery infusion chemotherapy(HAIC), interferon(IFN), internal radiotherapy(IRT), oral chemotherapy(OCT), transarterial chemoembolization(TACE)(Chen et al. 2021; Huo et al. 2020; Liu et al. 2021). However, there is still no consensus on the adjuvant treatment for patients with HCC after resection. Sorafenib(SOR) was not an effective adjuvant therapy for HCC following resection or ablation based on a large random controlled trial(RCT)(Bruix et al. 2015), and a post-hoc study identified that no mutation, gene amplification or proposed gene signatures predicted adjuvant sorafenib benefit(Pinyol et al. 2019). A meta-analysis recruited 7 studies, although most of them were retrospective studies, demonstrated that adjuvant TACE is superior for the patients with microvascular invasion (MVI)(Shen et al. 2020). However, another meta-analysis concluded that TACE, radiotherapy and sorafenib were listed as the beneficial treatment to prevent recurrence, although the studies included were mostly retrospective studies(Yang et al. 2021). HAIC was also identified as an option for adjuvant therapy, which had favorable prognosis on OS and disease-free survival (DFS) when compared with control group(Ke et al. 2021; Li et al. 2021). Other attempts such as OCT including 5-fluorouracil or uracil-tegafur(Hasegawa et al. 2006; Yamamoto et al. 1996), adoptive immunotherapy with lymphocyte infusions or cytokine-induced killer cells(Hui et al. 2009; Takayama et al. 2000), IFN(Nishiguchi et al. 2005), oral Huaier granule(Chen et al. 2018), et al. were identified as effective therapy for postoperative treatment.

However, there are still limitations for us to get a better conclusion of adjuvant therapy for these patients. One is the limited RCTs to verify the effectiveness of treatments, which needs more high quality studies to be carried out, another is deficiency of comparison between different options, which could be the best choice for the prevention of recurrence for the patients with HCC after curative resection. Herein, we try to collect all of the RCTs about adjuvant therapy for HCC after curative resection, and conclude the different effectiveness of variable adjuvant treatments by network meta-analysis(NMA).

## Methods

This study followed the Preferred Reporting Items for Systematic Review and Meta-Analyses (PRISMA) statement. As current meta-analysis was not based on individual patient-level, informed consent was waived.

### Search strategies and selection criteria

We searched Pubmed, Web of Science and Cochrane Library from database inception up through February 2023 for abstracts and full-text articles published about the comparison of different adjuvant procedures for patients with HCC after curative resection. Key words for the data search included liver cancer, hepatocellular carcinoma, adjuvant, post-operative. Consensus-based discussions were taken to solve the disagreements between authors(YYY and YW).

Studies including random controlled trials that compared the efficacy of different treatments for prevention of recurrence and acquisition of survival were selected. We excluded single arm studies or non-RCTs. We chose the most recent or complete study when duplicate publications or studies published in the same center with patients overlapped.

Two reviewers(YW and FMY) of us independently evaluated and extracted data from each study. The basic information of studies included: author/publication year, country, group, patients number, sex distribution, age, tumor size, tumor number, liver cirrhosis status, hepatitis status, Child-pugh score, Eastern Cooperative Oncology(ECOG), pre-operative AFP, macrovascular invasive, microvascular invasive and surgical margin. The primary outcome was the recurrence rate between different procedures. The secondary outcome was the survival of different treatments, including one-year survival, three-year survival and five-year survival.

### Risk of bias evaluation

The Cochrane risk of bias tool was used to evaluate the quality, which included the following domains: random sequence generation, allocation concealment, blinding, incomplete outcome data, and selective outcome reporting(Higgins et al. 2011). Two authors(HQX and FMY) evaluated the studies independently and made a consensus after discussion.

### Statistical analysis

The statistical analysis was conducted using Stata software (version 16, Stata Corp. LP, College station, TX, USA). Review Manager 5.3 software (Cochrane Collaboration, Oxford, UK) was used to evaluate the risk bias. The heterogeneity of direct evidence and in-direct evidence was according to inconsistency factor and the value of heterogeneity. The assessment of heterogeneity was according to the *I*^2^ test, and cut-off values of less than 25%, 25–75%, and greater than 75% represented low, moderate, and high heterogeneity, respectively. Network meta-analyses(NMA) of different treatments were using a random-effects models. League tables were generated for back-transformed network estimates. Odds ratios (ORs) and 95% confidence intervals were used to compare different treatments.

## Results

### Study selection and characteristics

**Fig. 1:**
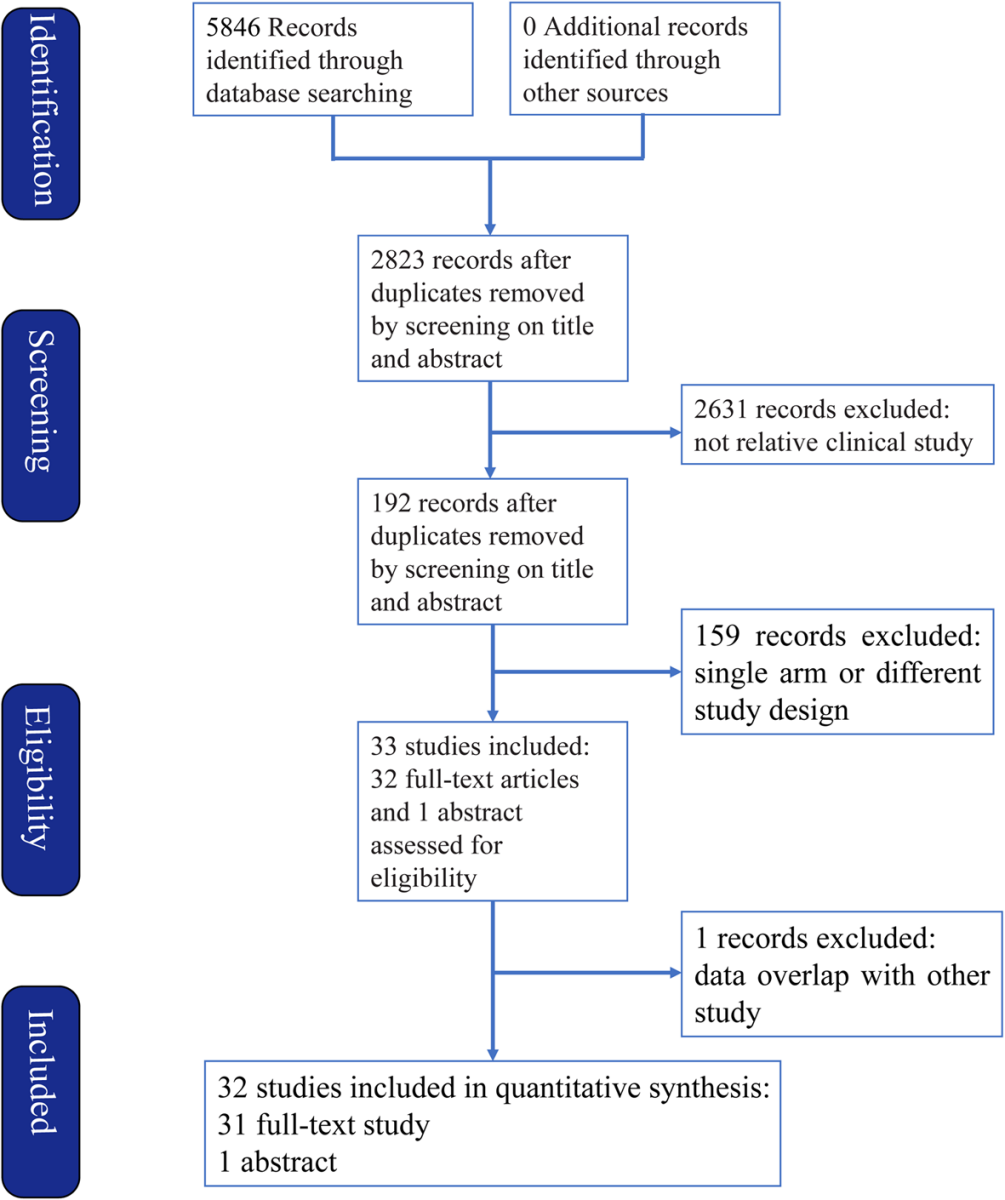
PRISMA Flow diagram of screening and selection strategy

Thirty-two studies(Bruix et al. 2015; Chen et al. 2012, 2013, 2018; Chung et al. 2013; Hachiya et al. 2020; Hasegawa et al. 2006; Hirokawa et al. 2020; Huang et al. 2015; Hui et al. 2009; Izumi et al. 1994; Lai et al. 1998; Lau et al. 1999; Li et al. 1995, 2006, 2020a, b; Lo et al. 2007; Matsui et al. 2021; Mazzaferro et al. 2006; Nishiguchi et al. 2005; Peng et al. 2009; Shi et al. 2022; Sun et al. 2006; Takayama et al. 2000; Wang et al. 2018; Wei et al. 2018; Xia et al. 2010; Xu et al. 2016; Yamamoto et al. 1996; Yu et al. 2014; Zhong et al. 2009) including 5783 patients were selected from a total of 5846 records(Fig. 1). The comparison of the studies included: ERT vs. non-adjuvant(NA)(*n* = 2); branched-chain amino acids(BCAA) vs. NA(*n* = 1); IRT vs. NA(*n* = 4); HAIC vs. NA(*n* = 3); dendritic cell(DC) vs. NA(*n* = 1); cytokine-induced killer(CIK) vs. NA(*n* = 2); AIT vs. NA(*n* = 1); Huaier vs. NA(*n* = 1); IFN vs. NA(*n* = 5); OCT vs. NA(*n* = 3); TACE vs. NA(*n* = 7); TACE vs. TACE + portal vein chemotherapy(PVC) vs. NA(*n* = 1); SOR vs. NA(*n* = 1). All of the studies included were random controlled trials, with matched number of patients, sex distribution, tumor size, liver cirrhosis status, Child-pugh score, ECOG, pre-operative alpha-fetoprotein(AFP) in each trial. However, there were some selection bias between different trials, such as macro-vascular invasive, micro-vascular invasive and surgical margin, which might be the most important factors that impact the tumor recurrence. The details of the studies were presented in Table 1.

**Table 1:** Baseline characteristics for patients included

**Author/publication year**	**Country**	**Group**	**Number**	**Sex(M/F)**	**Age(years old)**	**Tumor size(cm)**	**Tumor number**	**With liver cirrhosis**
Shi C, et al. 2022	China	ERT(SBRT)	38	(33/5)	56.42 ± 10.44	4.87 ± 2.03	1(1-3)	16(42.1%)
		NA	38	(32/6)	55.74 ± 10.19	4.88 ± 2.46	1(1-3)	15(39.5%)
Hachiya H, et al. 2020	Japan	BCAA	74	(59/15)	69(47-85)	2.7(1.0-15.9)	1:70(95%) ≥ 2:4(5%)	31(42%)
		NA	80	(66/14)	70(47-85)	2.9(0.8-12.0)	1:64(80%) ≥ 2:16(20%)	46(57%)
Li J, et al. 2020	China	IRT(^131^I-metuximab)	78	(58/20)	53.0(47.0-58.8)	4.9(3.2-6.4)	1:73(94%) > 1:5(6%)	42(54%)
		NA	78	(61/17)	53.0(47.2-58.0)	5.3(3.2-7.3)	1:74(95%) > 1:4(5%)	45(58%)
Li S, et al. 2020	China	HAIC	58	(52/6)	54(25-69)	5.8 ± 0.4	1:36(62.1%) ≥ 2:22(37.9%)	32(55.2%)
		NA	58	(49/9)	55.6 ± 1.6	5.5(1.8-16.0)	1:42(72.4%) ≥ 2:16(27.6%)	35(60.3%)
Hirokawa F, et al. 2020	Japan	HAIC	55	(48/7)	69(46-85)	3.4(1.0-14.5)	1:41(75%) ≥ 2:14(25%)	29(53%)
		NA	59	(48/11)	72(42-82)	3.1(1.0-12)	1:51(86%) ≥ 2:8(14%)	35(59%)
Matsui HM, et al. 2021	Japan	DC	30	(20/10)	72.5(36-81)	3.0(1.0-13.0)	1:21(70.0%) 2:6(20.0%) ≥ 3:3(10.0%)	10(33.3%)
		NA	14	(9/5)	70.0(57-85)	2.9(1.5-11.0)	1:10(71.4%) 2:4(28.6%) ≥ 3:0(0%)	2(14.3%)
Hui D, et al. 2009	China	CIK 3 courses	41	(31/10)	≥ 50:27 < 50:14	≥ 5:17 < 5:24	1:41(100%)	34(82.9%)
		CIK 6 courses	43	(32/11)	≥ 50:26 < 50:17	≥ 5:19 < 5:24	1:43(100%)	34(79.1%)
		NA	43	(34/9)	≥ 50:28 < 50:15	≥ 5:21 < 5:22	1:43(100%)	33(76.7%)
Takayama T, et al. 2000	Japan	AIT	76	NR	≥ 60:50(66%) < 60:26(34%)	< 3:38(50%) ≥ 3:38(50%)	1:51(67%) ≥ 2:25(33%)	35(46%)
		NA	74	NR	≥ 60:42(57%) < 60:32(43%)	<3:32(43%) ≥ 3:42(57%)	1:53(72%) ≥ 2:21(28%)	38(51%)
Xu L, et al. 2016	China	CIK	100	(92/8)	43(38-56)	≤ 3:30(30.0%) 3-5:35(35.0%) ≥ 5:35(35.0%)	1:95(95.0%) ≥ 2:5(5.0%)	55(55.0%)
		NA	100	(89/11)	52(43-60)	≤ 3:18(18.0%) 3-5:33(33.0%) ≥ 5:49(49.0%)	1:94(94.0%) ≥ 2:6(6.0%)	58(58.0%)
Yu W, et al.^2014^	China	ERT	58	(51/7)	53.1 ± 10.5	4.7 ± 2.6	1:52(89.7%) 2:6(10.3%)	51(87.9%)
		NA	61	(48/13)	55.5 ± 10.7	5.6 ± 3.7	1:53(86.9%) 2:8(13.1%)	54(88.5%)
Huang SX, et al. 2015	China	HAIC	42	(31/11)	59.1±6.2	6.2±1.5	1:24(57.1%) ≥ 2 :18(42.9%)	NR
		NA	43	(30/13)	58.4±5.7	5.7±1.3	1:23(53.5%) ≥ 2:20(46.5%)	NR
Chen Q, et al. 2018	China	Huaier	686	(565/121)	< 65:573(83.53%) ≥ 65:113(16.47%)	< 2:55(8.02%) ≥ 2, < 5:340(49.56%) ≥ 5, < 10:240(34.99%) ≥ 10:51(7.43%)	1:595(86.73%) ≥ 2 :91(13.27%)	473(68.95%)
		NA	316	(255/61)	< 65:275(87.03%) ≥ 65:41(12.97%)	< 2:25(7.91%) ≥ 2,< 5:149(47.15%) ≥ 5, < 10:100(31.65%) ≥ 10:42(13.29%)	1:274(86.71%) ≥ 2 :42(13.29%)	198(62.66%)
Chen LT, et al. 2012	China	IFN	133	(108/25)	50(48-54)	3.5(3.0-4.0)	1:103(77.4%) ≥ 2:30(22.6%)	73(54.9%)
		NA	135	(112/23)	49(46-51)	3.0(2.5-3.5)	1:115(85.2%) ≥ 2:20(14.8%)	74(54.8%)
Lo CM, et al. 2007	China	IFN	40	(31/9)	49(26-75)	5.5(1.8-22)	1:33(83%) ≥ 2:7(17%)	19(48%)
		NA	40	(34/6)	54(24-74)	5.7(1.2-18)	1:29(73%) ≥ 2:11(27%)	19(48%)
Mazzaferro V, et al. 2006	Italy	IFN	76	(61/15)	65(41-74)	3.37 ± 2.75(0.3-19)	1:59(77.6%) ≥ 2 :17(22.4%)	NR
		NA	74	(51/23)	67(36-73)	3.19 ± 2.26(0.3-14.5)	1:55(74.3%) ≥ 2:19(25.7%)	NR
Nishiguchi S, et al. 2005	Japan	IFN	15	(15/0)	61.9 ± 5.8	2.5(1.9-3.5)	NR	NR
		NA	15	(15/0)	60.0 ± 4.8	2.6(2.4-3.5)	NR	NR
Sun HC, et al. 2006	China	IFN	118	(106/12)	52.2	4.3±2.7	1:102(86.4%) >1:16(13.6%)	98(83.1%)
		NA	118	(102/16)	50.4	4.9±3.0	1:103(87.3%) ≥ 2 :15(12.7%)	104(88.1%)
Chen K, et al. 2013	China	IRT(^125^I)	34	(25/9)	50.79 ± 6.79	6.24 ± 2.55	1:30(88.2%) ≥ 2:4(11.8%)	18(52.9%)
		NA	34	(24/10)	48.91 ± 7.30	5.65±2.52	1:31(91.2%) ≥ 2:3(8.8%)	20(58.8%)
Chung AY, et al. 2013	Singapore	IRT(^131^I-L)	51	(41/10)	65(22-82)	4.2(0.4-30.0)	1:49(96.1%) ≥ 2:2(3.9%)	NR
		NA	52	(45/7)	63(42-84)	3.8(1.4-18.0)	1:43(82.7%) ≥ 2 :9(17.3%)	NR
Lau WY, et al. 1999	China	IRT(^131^I-L)	21	(17/4)	51(23-71)	4.4(1.4-11)	1:14(66.7%) ≥ 2 :7(33.3%)	NR
		NA	22	(18/4)	54(24-75)	3.8(1.5-10)	1:18(81.8%) ≥ 2:4(18.2%)	NR
Hasegawa K, et al. 2006	Japan	OCT(UFT)	79	(60/19)	65(29-75)	3.3(1.2-12)	1:53(67.1%) ≥ 2:26(32.9%)	42(53.2%)
		NA	80	(65/15)	64(35-78)	3.4(0.7-13)	1:58(72.5%) ≥ 2 :22(27.5%)	38(47.5%)
Xia Y, et al. 2010	China	OCT（capecitabine）	30	(25/5)	≤ 60:27(90.0%) > 60:3(10.0%)	7.27±4.37	1:25(83.3%) ≥2:5(16.7%)	19(63.3%)
		NA	30	(21/9)	≤ 60:24(80.0%) > 60:6(20.0%)	6.34±3.16	1:26(86.7%) ≥2:4(13.3%)	21(70.0%)
Yamamoto M, et al. 1996	Japan	OCT(HCFU)	28	NR	NR	NR	NR	31(65%)
		NA	27	NR	NR	NR	NR	10(83.3%)
Peng BG, et al. 2009	China	TACE	51	(46/5)	46.2±13.8	9.04±3.02	≤ 3 (100%)	42(82%)
		NA	53	(50/3)	50.2±7.5	8.39±2.29	≤ 3 (100%)	37(70%)
Wang Z, et al. 2018	China	TACE	140	(121/19)	54.2±9.7	≤ 5:56(40.0%) > 5:84(60.0%)	1：102(72.9%) ≥ 2:38(27.1%)	72(51.4%)
		NA	140	(109/31)	52.6±10.3	≤ 5:61(43.6%) > 5:79(56.4%)	1:109(77.9%) ≥2:31(22.1%)	66(47.1%)
Wei W, et al. 2018	China.	TACE	116	(106/10)	44.0(18-75)	5-10:82(70.7%) > 10:34(29.3%)	1:116(100%)	50(43.1%)
		NA	118	(106/12)	48.5(18-74)	5-10:97(82.2%) > 10:21(17.8%)	1:118(100%)	42(35.6%)
Zhong C, et al. 2009	China	TACE	57	(53/4)	47.6±10.4	9.5±3.8	1:13(22.8%) ≥2:44(77.2%)	50(87.7%)
		NA	58	(49/9)	48.2±11.2	9.7±3.6	1:16(27.6%) ≥2:42(72.4%)	48(82.8%)
Li Q, et al. 2006	China	TACE	39	(34/5)	52.5±11.4	5.3 ± 2.1	1:33(84.6%) ≥2:6(15.4%)	NR
		TACE+PVC	47	(38/9)	48.6±11.0	5.0 ± 1.9	1:35(74.5%) ≥2:12(25.5%)	NR
		NA	45	(39/6)	50.9 ± 9.9	5.1 ± 1.3	1:36(80.0%) ≥2:9(20.0%)	NR
Li JQ, et al. 1995	China	TACE	47	NR	NR	< 5:30 5-10:76 > 10:34	NR	Severe:8 Moderate:38 Mild:62 No:32
		NA	47	NR	NR		NR	
Lai EC, et al. 1998	China	TACE	30	(26/4)	54.6(50.2-59)	8.5(6.8-10.1)	1:19(63.3%) ≥2:11(36.7%)	17(56.7%)
		NA	36	(27/9)	53.4(49.2-57.5)	10.4(5.2-15.6)	1:21(58.3%) ≥2:15(41.7%)	19(52.8%)
Izumi R, et al. 1994	Japan	TACE	23	(21/2)	62.1 ± 8.9	≤5:8(34.8%) >5:15(65.2%)	1:6(26.1%) ≥2:17(73.9%)	19(82.6%)
		NA	27	(23/4)	64.8 ± 10.6	≤5:4(14.8%) >5:23(85.2%)	1:16(59.3%) ≥2:11(40.7%)	22(81.5%)
Bruix J, et al. 2015	Spain,Japan,Italy,China.	SOR	556	(451/105)	58(24-85)	3.5(1.0-20.0)	1:506(91%) 2:44(8%) ≥3:6(1%)	357(64%)
		NA	558	(461/97)	60(19-83)	3.5(1.0-19.0)	1:521(93%) 2:33(6%) ≥3:4(< 1%)	344(62%)

**Author/publication year**	**With ** **hepatitis** **（** **HBV** **/HCV** **）**	**Child-** **pugh** ** score**	**ECOG**	**Pre-operative AFP(ng/ml)**	**Macro-vascular invasive**	**Micro-vascular invasive**	**Surgical margin**
Shi C, et al. 2022	36(94.7%)	5-6:34(89.5%)	NR	AFP positive 21(55.3%)	NR	NR	22(57.9%) positive margins
	36(94.7%)	5-6:36(94.7%)	NR	AFP positive 25(65.8%)	NR	NR	24(63.2%) positive margins
Hachiya H, et al. 2020	59(80%)	A:61(82%) B:13(18%)	NR	9(2-10000)	PV:16(21%) HV:1(1%)	NR	NR
	67(83%)	A:64(80%) B:16(20%)	NR	12(2-6000)	PV:20(25%) HV:1(1%)	NR	NR
Li J, et al. 2020	HBV:66(85%) HCV:0	NR	NR	≤ 20 34(44%) 20-400 18(23%) ≥ 400 26(33%)	NR	31(40%)	Margin< 1cm :39 (50%) Margin ≥ 1cm:39(50%)
	HBV:60(76%) HCV:3(4%)	NR	NR	≤ 20 30(38%) 20-400 27(35%) ≥ 400 21(27%)	NR	32(41%)	Margin< 1cm:37(47%) Margin ≥ 1cm:41(53%)
Li S, et al. 2020	HBV:54(93.1%) HCV:2(3.4%)	5:57(98.3%) 6:1(1.7%)	≤ 2	176.7(0.87-121000)	NR	58(100.0%)	Negative margin
	HBV:51(87.9%) HCV:1(1.7%)	5:56(96.6%) 6:2(3.4%)	≤ 2	261.2(1.51-121000)	NR	58(100.0%)	Negative margin
Hirokawa F, et al. 2020	HBV/HCV:35(64%) HCV:21(38%)	A:49(89%) B:6(11%)	NR	9.4(2.1-141876)	NR	14(25%)	Median surgical margin:5(0-32)
	HBV/HCV:34(58%) HCV:24(41%)	A:51(86%) B:8(14%)	NR	9.8(2.1-138642)	NR	11(19%)	Median surgical margin:5(0-36)
Matsui HM, et al. 2021	HBV:1(3.3%) HCV:22(73.3%)	A:30(100%)	NR	274(117-483)	NR	9(30.0%)	NR
	HBV:2(14.3%) HCV:7(50.0%)	A:14(100%)	NR	282(116-471)	NR	4(28.6%)	NR
Hui D, et al.^2009^	HBV:32(78.0%)	A:34(82.9%) B:7(17.1%)	NR	AFP positive 33(80.5%)	NR	17(41.5%)	Resection margin >1cm
	HBV:33(76.7%)	A:34(79.1%) B:9(20.9%)	NR	AFP positive 34(79.1%)	NR	19(44.2%)	Resection margin >1cm
	HBV:31(72.1%)	A:34(79.1%) B:9(20.9%)	NR	AFP positive 33(76.7%)	NR	23(53.5%)	Resection margin >1cm
Takayama T, et al. 2000	HBV:15(20%) HCV:50(66%)	A:54(71%) B:22(29%)	NR	< 400:58(76%) ≥ 400:18(24%)	NR	34(45%)	NR
	HBV:14(19%) HCV:49(66%)	A:50(68%) B:24(32%)	NR	< 400:57(77%) ≥ 400:17(23%)	NR	32(43%)	NR
Xu L, et al. 2016	HBV:84(84.0%)	A:100(100.0%)	0:89(89.0%)1:11(11.0%)	< 25:44(44.0%) 25-400:24(24.0%) 400-10000:23(23.0%) > 10000:9(9.0%)	NR	2(2.0%)	Negative margin
	HBV:87(87.0%)	A:100(100.0%)	0:88(88.0%)1:12(12.0%)	< 25:50(50.0%) 25-400:13(13.0%) 400-10000:25(25.0%) > 10000:12(12.0%)	NR	1(1.0%)	Negative margin
Yu W, et al. 2014	HBV:52(89.7%) HCV:1(1.7%)	A:58(100.0%)	NR	> 25:24(41.4%) ≤ 25:34(58.6%)	PV adhesion:12(20.7%) PV+HV adhesion:10(17.2%) HV adhesion:18(31.0%)	7(12.1%)	Margin ≤ 1cm:58(100.0%)
	HBV:53(86.9%) HCV:5(8.2%)	A:61(100.0%)	NR	> 25:27(44.3%) ≤ 25:34(55.7%)	PV adhesion:12(19.7%) PV+HV adhesion:9(14.8%) HV adhesion:25(41.0%)	8(13.1%)	Margin≤ 1cm:61(100.0%)
Huang SX, et al. 2015	NR	A:24(57.1%) B:18(42.9%)	0-2(100%)	562.4 ± 54.1	NR	NR	NR
	NR	A:27(62.8%) B:16(37.2%)	0-2(100%)	547.5 ± 49.2	NR	NR	NR
Chen Q, et al. 2018	HBV:544(79.30%)HCV:8(1.17%)	A:643(93.73%)B:43(6.27%)	NR	<400:465(67.98%) ≥400:219(32.02%)	NR	NR	Margin ≥ 1cm
	HBV:234(74.05%) HCV:5(1.58%)	A:291(92.09%) B:25(7.91%)	NR	<400:201(64.42%) ≥400:111(35.58%)	NR	NR	Margin ≥ 1cm
Chen LT, et al. 2012	HBV:106(79.7%) HCV:27(20.3%)	≤ 7:133(100.0%)	0:75(56.4%) ≥ 1:58(43.6%)	≥ 100:16(12.0%) < 100:117(88.0%)	NR	41(30.8%)	Margin ≥ 1cm and negative
	HBV:108(90.0%) HCV:26(19.2%)	≤ 7:135(100.0%)	0:82(60.7%) ≥ 1:53(39.3%)	≥ 100:17(12.6%) < 100:118(87.4%)	NR	33(24.4%)	Margin ≥ 1cm and negative
Lo CM, et al. 2007	HBV:38(95%) HCV:1(3%)	NR	NR	126(2-182900)	19(48%)	NR	0(0%)
	HBV:39(98%) HCV:2(5%)	NR	NR	23(3-103540)	14(35%)	NR	0(0%)
Mazzaferro V, et al. 2006	HCV:76(100.0%)	A:70(92.1%) B:6(7.9%)	NR	16(2-6854)	NR	15(19.7%)	Margin<1cm:37(53.6%) Margin≥1cm:32(46.4%)
	HCV:74(100.0%)	A:70(94.6%) B:4(5.4%)	NR	20(1-6648)	NR	17(23.0%)	Margin<1cm:34(50.0%) Margin≥1cm:34(50.0%)
Nishiguchi S, et al. 2^005^	HCV:15(100.0%)	A:11(73.3%) B:4(26.7%)	NR	> 100:4(26.7%)	NR	NR	NR
	HCV:15(100.0%)	A:12(80.0%) B:3(20.0%)	NR	> 100:4(26.7%)	NR	NR	NR
Sun HC, et al. 2006	HBV:118(100.0%)	NR	NR	≤ 20:47(39.8%) > 20:71(60.2%)	NR	90(76.3%)	NR
	HBV:118(100.0%)	NR	NR	≤ 20:36(30.5%) > 20:82(69.5%)	NR	90(75.4%)	NR
Chen K, et al. 2013	HBV:26(76.5%) HCV:6(17.6%)	A:34(100.0%)	NR	611.97 ± 265.94	NR	17(50.0%)	Margin<2cm:5(14.7%) Margin≥2cm:29(85.3%)
	HBV:31(91.2%) HCV:5(14.7%)	A:34(100.0%)	NR	579.26 ± 298.46	NR	14(41.2%)	Margin<2cm:5(14.7%) Margin≥2cm:29(85.3%)
Chung AY, et al. 2013	HBV:29(56.9%)	NR	NR	29.3(1.0-70700)	PV:1(2.0%) HV:2(3.9%)	15(29.4%)	Clearly Margin ≥ 1mm
	HBV:32(61.5%)	NR	NR	13.1(1.3-2774)	PV:2(3.9%) HV:1(1.9%)	14(26.9%)	Clearly Margin≥1mm
Lau WY, et al. 1999	HBV:19(90.5%)	NR	NR	147(4-13300)	NR	1(4.8%)	Clear resection margin≥1cm
	HBV:19(86.4%)	NR	NR	213(3-27170)	NR	1(4.5%)	Clear resection margin≥1cm
Hasegawa K, et al. 2006	HBV:14(17.7%) HCV:58(73.4%)	A:68(86.1%) B:11(13.9%)	NR	29(2-49715)	NR	18(22.8%)	NR
	HBV:15(18.8%) HCV:56(70%)	A:70(87.5%) B:10(12.5%)	NR	29(1-49388)	NR	17(21.3%)	NR
Xia Y, et al. 2010	HBV:26(86.7%)	A:30(100.0%)	NR	<400:20(66.7%) ≥400:10(33.3%)	NR	18(60.0%)	Tumor-free resectionmargin≥ 1cm
	HBV:24(80.0%)	A:30(100.0%)	NR	<400:15(50.0%)≥400:15(50.0%)	NR	20(66.7%)	Tumor-free resectionmargin≥ 1cm
Yamamoto M, et al. 1996	NR	NR	NR	NR	PV:1(1.8%)	NR	Margin≥1cm
	NR	NR	NR	NR	NR	NR	Margin≥1cm
Peng BG, et al. 2009	HBV:31(61%)HCV:5(10%)	A:44(86.3%) B:7(13.7%)	NR	<400:20(39%)≥400:31(61%)	PV vp1 or vp2:18(35%)vp3:22(43%)vp4:11(22%)	NR	Margin≥ 2cm
	HBV:40(76)HCV:3(6%)	A:46(86.8%) B:7(13.2%)	NR	<400:15(28%)≥400:38(72%)	PV vp1 or vp2:18(35%)vp3:22(43%)vp4:11(22%)	NR	Margin≥ 2cm
Wang Z, et al. 2018	HBV:140(100%)HCV:0(0.0%)	Child A/B(100%)	NR	≤20:53(37.9%)>20:87(62.1%)	NR	78(55.7%)	Clear margin
	HBV:140(100%)HCV:0(0.0%)	Child A/B(100%)	NR	≤20:51(36.4%)>20:89(63.6%)	NR	87(62.1%)	Clear margin
Wei W, et al. 2018	94(81.0%)	A:116(100.0%) B:0(0.0%)	0:48(41.4%) 1:65(56.0%) 2:3(2.6%)	< 25:37(31.9%) ≥ 25:79(68.1%)	NR	NR	Margin<2cm:91(78.4%) Margin≥2cm:25(21.6%)
	101(85.6%)	A:116(98.3%) B:2(1.7%)	0:53(44.9%) 1:63(53.4%) 2:2(1.7%)	< 25:36(30.5%) ≥ 25:82(69.5%)	NR	NR	Margin<2cm:92(78.0%) Margin≥2cm:26(22.0%)
Zhong C, et al. 2009	HBV:53(93.0%)	A:56(98.2%) B:1(1.8%)	NR	>25:41(71.9%) ≤25:16(28.1%)	PV vp1 or vp2:15(26.3%) vp3 or vp4:7(12.3%) HV:1(1.8%)	NR	1.4 ± 0.8cm
	HBV:52(89.7%)	A:58(100.0%) B:0(0.0%)	NR	>25:45(77.6%) ≤25:13(22.4%)	PV vp1 or vp2:14(24.1%) vp3 or vp4:9(15.5%); HV:2(3.4%)	NR	1.1 ± 0.7cm
Li Q, et al. 2006	HBV:32(82.1%)	A:23(59.0%) B:16(41.0%)	NR	590.1 ± 583.1	NR	NR	NR
	HBV:40(85.1%)	A:28(59.6%) B:19(40.4%)	NR	580.9 ± 496.1	NR	NR	NR
	HBV:37(82.2%)	A:22(48.9%) B:23(51.1%)	NR	470.9 ± 399.8	NR	NR	NR
Li JQ, et al. 1995	NR	NR	NR	NR	NR	NR	NR
	NR	NR	NR	NR	NR	NR	NR
Lai EC, et al. 1998	HBV:25(83.3%)	NR	NR	246.5(1-735000)	14(46.7%)	NR	Median resection margin:1.39(95%CI:0.96-1.82)cm; Positive margin:1(3.3%)
	HBV:31(86.1%)	NR	NR	181.0(1-388800)	16(44.4%)	NR	Median resection margin:1.45(95%CI:1-1.87)cm; Positive margin:5(13.9%)
Izumi R, et al. 1994	HBV:6(26.1%) HCV:3(13.0%)	NR	NR	≤ 200:7(30.4%) > 200:16(69.6%)	PV:13(56.5%) HV:9(39.1%)	NR	NR
	HBV:2(7.4%) HCV:3(11.1%)	NR	NR	≤ 200:9(33.3%)> 200:18(66.7%)	PV:14(51.9%) HV:14(51.9%)	NR	NR
Bruix J, et al. 2015	HBV:282(51%) HCV:119(21%)	5:429(77%) 6:112(20%) 7:15(3%) 8:0	0:551(99%) 1:5(< 1%)	6.0(1.1-348.4)	NR	146(32%)	Resection R0
	HBV:264(47%) HCV:151(27%)	5:432(77%) 6:106(19%) 7:16(3%) 8:4(< 1%)	0:555(100%) 1:3(< 1%)	5.6(1.0-532.8)	NR	147(33%)	Resection R0

*ERT *External radiotherapy, *BCAA *Branched-chain amino acids, *IRT *Internal radiotherapy, *HAIC *Hepatic artery infusion chemotherapy, *DC *Dendritic cell, *CIK *Cytokine-induced killer, *AIT *Adoptive immunotherapy, *IFN *Interferon, *OCT *Oral chemotherapy, *TACE *Transcatheter arterial chemoembolization, *PVC *Portal vein chemotherapy, *SOR *Sorafenib, *NA *Non-adjuvant, *NR *No report, *SBRT *Stereotactic body radiotherapy, *ECOG *Eastern Cooperative Oncology Group, *HV *Hepatic vein, *PV *Portal vein

**Fig. 2:**
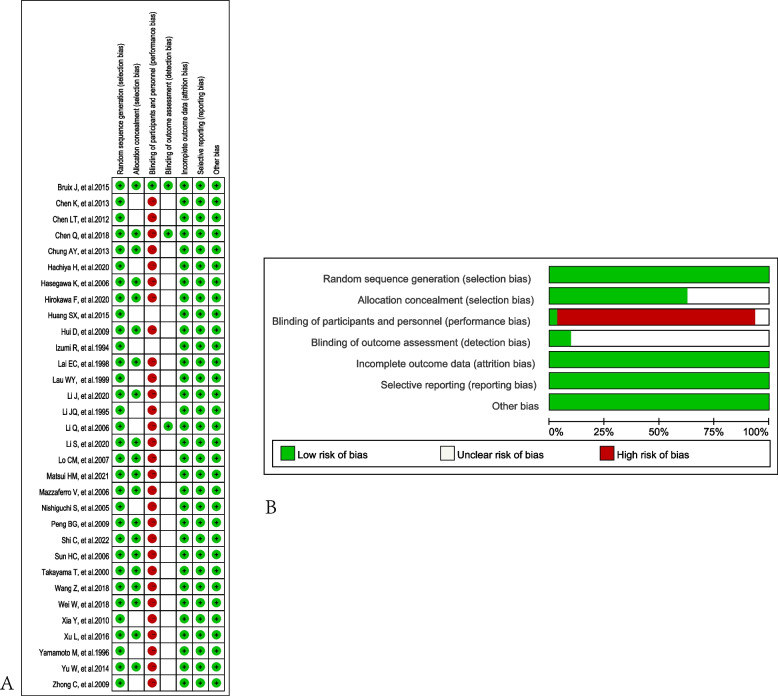
RCTs bias evaluated by Cochrane risk of bias tool

When we considered the risk of bias, most of the studies have perfect random sequence generation, allocation concealment, complete outcome data and low reporting bias, whereas the different treatments correlated with different procedures or adverse reactions that were easy to distinguish with non-adjuvant therapy cause the impossible for blinding of participants and personnel for nearly all trials. Moreover, there were a small number of studies reported blinding of outcome assessment. The risk of bias was presented in Fig. 2.

### Network meta-analysis

**Fig. 3:**
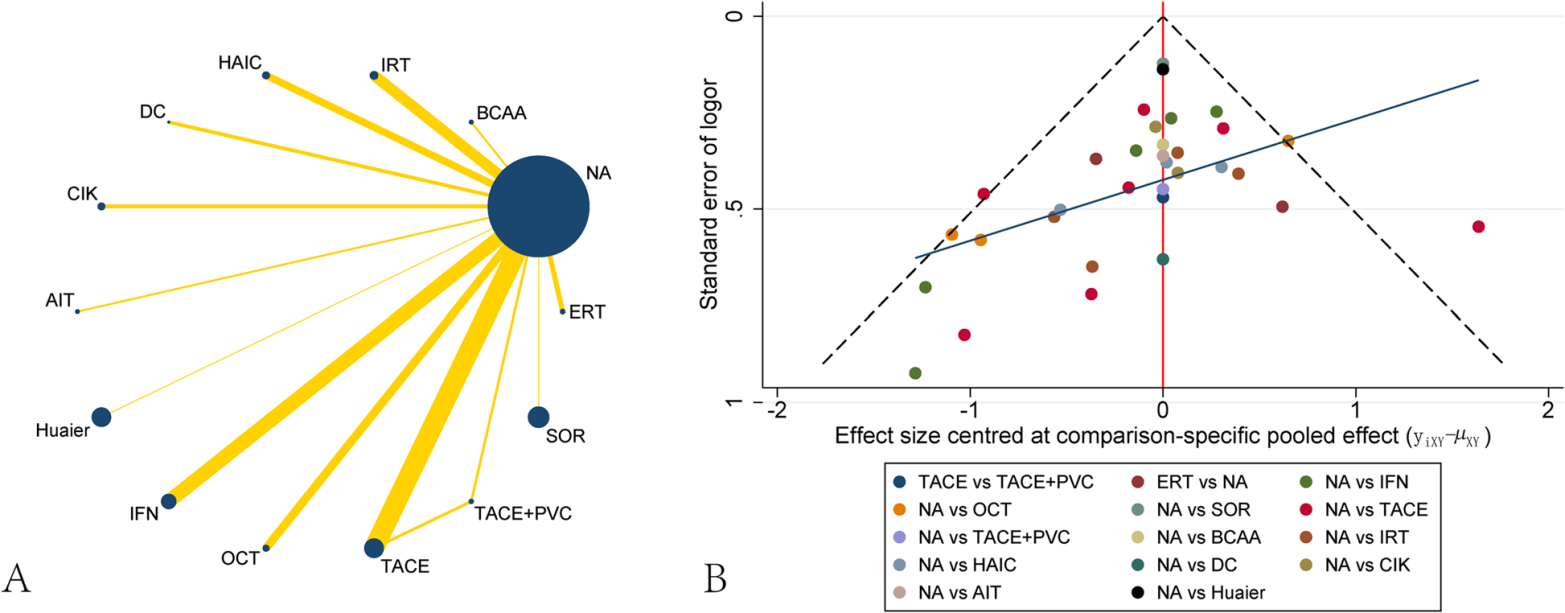
Network plot and funnel plot of studies included in the analysis of recurrence. **A** Network plot of studies included in the analysis of recurrence. **B** funnel plot of studies included in the analysis of recurrence. ERT, external radiotherapy; BCAA, branched-chain amino acids; IRT, internal radiotherapy; HAIC, hepatic artery infusion chemotherapy; DC, dendritic cell; CIK, cytokine-induced killer; AIT, adoptive immunotherapy; IFN, interferon; OCT, oral chemotherapy; TACE, transcatheter arterial chemoembolization; PVC, portal vein chemotherapy; SOR, sorafenib; NA, non-adjuvant

Thirty-one studies including 5629 patients were collected for preventing recurrence between different treatments. The network plot showed that most of the studies were compared different treatments with NA, whereas there was a loop between TACE, TACE + PVC and NA(Fig. 3A). The funnel plot also demonstrated uniform distribution of different comparisons(Fig. 3B). The network analysis showed benefit from TACE + PVC[OR, 2.84 (1.15,6.99)] and IRT[OR, 2.63 (1.41,4.91)] when considering recurrence. SOR[OR, 0.57 (0.24,1.38)] had the trend of increasing recurrence rate, although there were no significant differences between them. Other treatments including TACE, OCT, IFN, Huaier, HAIC, ERT, CIK, BCAA and AIT didn’t present significant difference with NA, although all of them had the trend of preventing recurrence(Table 2). The ranking of different treatments according to surface under the cumulative ranking curve (SUCRA) was as follows: TACE + PVC, 88.0%; IRT, 82.3%; ERT, 72.8%; AIT, 71.8%; Huaier, 59.4%; HAIC, 58.7%; TACE, 51.0%; IFN, 48.0%; OCT, 39.0%; CIK, 36.7%; DC, 30.7%; BCAA, 30.5%; NA, 23.4%; SOR, 7.8%(Table 3).

**Table 2:** League table of network meta-analysis for preventing recurrence

TACE	2.53 (0.94,6.81)	0.51 (0.21,1.26)	1.19 (0.50,2.82)	1.45 (0.92,2.27)	0.55 (0.25,1.19)	1.02 (0.50,2.08)	0.84 (0.31,2.27)	0.86 (0.38,1.94)	0.65 (0.25,1.70)	1.55 (0.32,7.40)	1.24 (0.51,3.01)	1.42 (0.45,4.55)	0.63 (0.19,2.06)
0.40 (0.15,1.06)	SOR	0.20 (0.06,0.71)	0.47 (0.15,1.48)	0.57 (0.24,1.38)	0.22 (0.07,0.64)	0.41 (0.14,1.14)	0.33 (0.09,1.16)	0.34 (0.11,1.03)	0.26 (0.08,0.87)	0.61 (0.11,3.48)	0.49 (0.15,1.57)	0.56 (0.14,2.25)	0.25 (0.06,1.02)
1.96 (0.80,4.85)	4.97 (1.41,17.51)	TACE + PVC	2.33 (0.73,7.48)	2.84 (1.15,6.99)	1.08 (0.36,3.23)	2.01 (0.70,5.78)	1.65 (0.46,5.84)	1.69 (0.54,5.22)	1.28 (0.37,4.40)	3.04 (0.53,17.46)	2.43 (0.75,7.93)	2.80 (0.69,11.33)	1.23 (0.30,5.11)
0.84 (0.35,2.01)	2.13 (0.67,6.75)	0.43 (0.13,1.38)	OCT	1.22 (0.58,2.56)	0.46 (0.18,1.22)	0.86 (0.35,2.12)	0.71 (0.22,2.25)	0.72 (0.26,1.98)	0.55 (0.18,1.68)	1.31 (0.25,6.95)	1.04 (0.36,3.04)	1.20 (0.33,4.42)	0.53 (0.14,2.00)
0.69 (0.44,1.09)	1.75 (0.73,4.22)	0.35 (0.14,0.87)	0.82 (0.39,1.73)	NA	0.38 (0.20,0.71)	0.71 (0.41,1.23)	0.58 (0.24,1.41)	0.59 (0.30,1.17)	0.45 (0.19,1.05)	1.07 (0.24,4.79)	0.86 (0.40,1.84)	0.99 (0.34,2.87)	0.43 (0.14,1.31)
1.82 (0.84,3.93)	4.60 (1.56,13.54)	0.93 (0.31,2.77)	2.16 (0.82,5.66)	2.63 (1.41,4.91)	IRT	1.86 (0.82,4.25)	1.53 (0.51,4.52)	1.56 (0.62,3.93)	1.19 (0.42,3.38)	2.82 (0.56,14.27)	2.25 (0.84,6.05)	2.59 (0.75,8.94)	1.14 (0.32,4.05)
0.98 (0.48,1.98)	2.47 (0.87,6.97)	0.50 (0.17,1.43)	1.16 (0.47,2.84)	1.41 (0.81,2.45)	0.54 (0.24,1.23)	IFN	0.82 (0.29,2.33)	0.84 (0.35,2.00)	0.64 (0.23,1.73)	1.51 (0.31,7.45)	1.21 (0.47,3.10)	1.39 (0.42,4.63)	0.61 (0.18,2.10)
1.19 (0.44,3.23)	3.01 (0.86,10.53)	0.61 (0.17,2.15)	1.41 (0.44,4.50)	1.72 (0.71,4.19)	0.66 (0.22,1.94)	1.22 (0.43,3.47)	Huaier	1.02 (0.33,3.14)	0.78 (0.23,2.65)	1.85 (0.32,10.53)	1.48 (0.46,4.77)	1.70 (0.42,6.82)	0.75 (0.18,3.08)
1.17 (0.51,2.64)	2.95 (0.97,8.98)	0.59 (0.19,1.84)	1.38 (0.51,3.78)	1.68 (0.85,3.33)	0.64 (0.25,1.62)	1.19 (0.50,2.86)	0.98 (0.32,3.00)	HAIC	0.76 (0.26,2.25)	1.81 (0.35,9.36)	1.44 (0.52,4.02)	1.66 (0.47,5.90)	0.73 (0.20,2.67)
1.53 (0.59,3.99)	3.88 (1.15,13.12)	0.78 (0.23,2.68)	1.82 (0.59,5.55)	2.22 (0.95,5.15)	0.84 (0.30,2.40)	1.57 (0.58,4.27)	1.29 (0.38,4.38)	1.31 (0.44,3.89)	ERT	2.37 (0.43,13.24)	1.90 (0.61,5.93)	2.18 (0.56,8.52)	0.96 (0.24,3.85)
0.65 (0.14,3.09)	1.63 (0.29,9.28)	0.33 (0.06,1.89)	0.77 (0.14,4.07)	0.93 (0.21,4.17)	0.36 (0.07,1.80)	0.66 (0.13,3.26)	0.54 (0.09,3.09)	0.55 (0.11,2.87)	0.42 (0.08,2.35)	DC	0.80 (0.15,4.30)	0.92 (0.15,5.79)	0.40 (0.06,2.60)
0.81 (0.33,1.96)	2.04 (0.64,6.56)	0.41 (0.13,1.34)	0.96 (0.33,2.79)	1.17 (0.54,2.51)	0.44 (0.17,1.19)	0.83 (0.32,2.12)	0.68 (0.21,2.19)	0.69 (0.25,1.93)	0.53 (0.17,1.65)	1.25 (0.23,6.72)	CIK	1.15 (0.31,4.28)	0.51 (0.13,1.94)
0.70 (0.22,2.24)	1.77 (0.44,7.09)	0.36 (0.09,1.45)	0.83 (0.23,3.06)	1.01 (0.35,2.95)	0.39 (0.11,1.33)	0.72 (0.22,2.39)	0.59 (0.15,2.36)	0.60 (0.17,2.14)	0.46 (0.12,1.79)	1.09 (0.17,6.84)	0.87 (0.23,3.24)	BCAA	0.44 (0.09,2.04)
1.60 (0.48,5.27)	4.04 (0.98,16.59)	0.81 (0.20,3.38)	1.89 (0.50,7.17)	2.31 (0.77,6.97)	0.88 (0.25,3.13)	1.64 (0.48,5.62)	1.34 (0.32,5.53)	1.37 (0.37,5.02)	1.04 (0.26,4.18)	2.47 (0.38,15.91)	1.98 (0.52,7.58)	2.28 (0.49,10.59)	AIT

*ERT *External radiotherapy, *BCAA *Branched-chain amino acids, *IRT *Internal radiotherapy, *HAIC *Hepatic artery infusion chemotherapy, *DC *Dendritic cell, *CIK *Cytokine-induced killer, *AIT *Adoptive immunotherapy, *IFN *Interferon, *OCT *Oral chemotherapy, *TACE *Transcatheter arterial chemoembolization, *PVC *Portal vein chemotherapy, *SOR *Sorafenib, *NA *Non-adjuvant

**Table 3:** Surface under the cumulative ranking curve (SUCRA) values for recurrence

Treatment	SUCRA(%)
TACE + PVC	88.0
IRT	82.3
ERT	72.8
AIT	71.8
Huaier	59.4
HAIC	58.7
TACE	51.0
IFN	48.0
OCT	39.0
CIK	36.7
DC	30.7
BCAA	30.5
NA	23.4
SOR	7.8

**Fig. 4:**
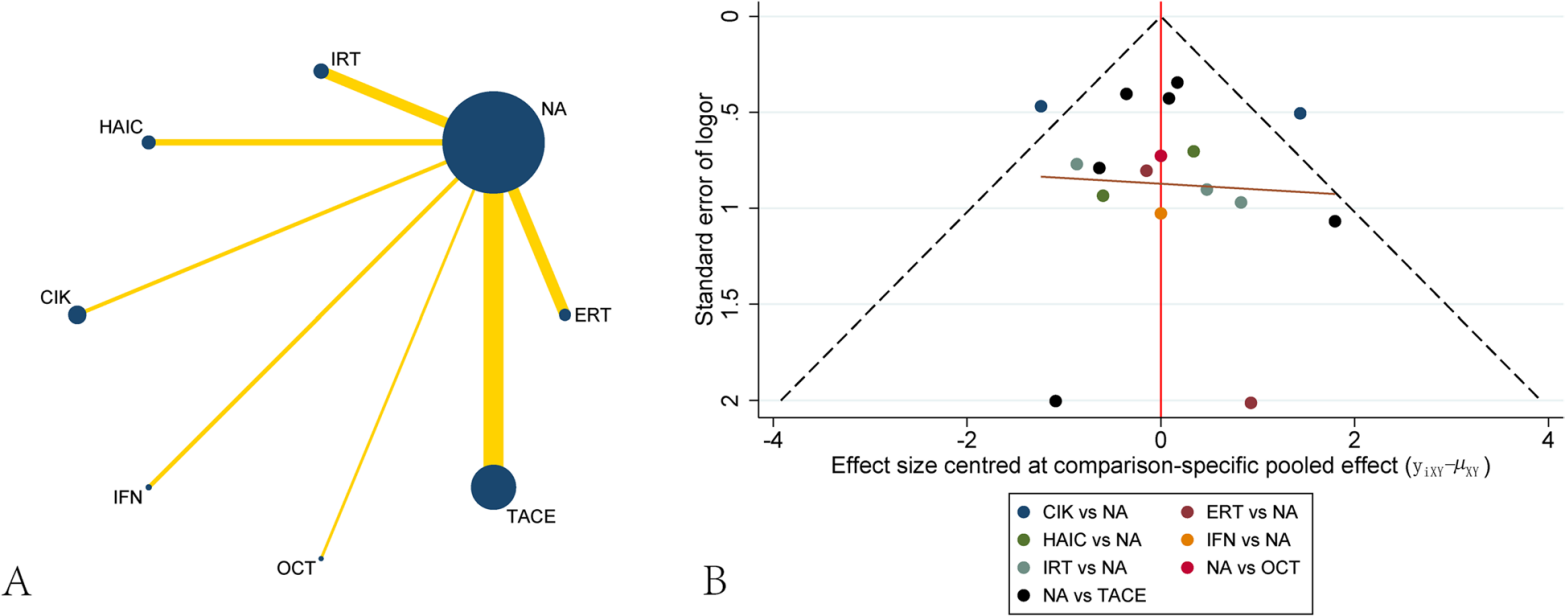
Network plot and funnel plot of studies included in the analysis of one-year survival. **A** Network plot of studies included in the analysis of one-year survival. **B** funnel plot of studies included in the analysis of one-year survival. ERT, external radiotherapy; IRT, internal radiotherapy; HAIC, hepatic artery infusion chemotherapy; CIK, cytokine-induced killer; IFN, interferon; OCT, oral chemotherapy; TACE, transcatheter arterial chemoembolization; NA, non-adjuvant

*ERT *External radiotherapy, *BCAA *Branched-chain amino acids, *IRT *Internal radiotherapy, *HAIC *Hepatic artery infusion chemotherapy, *DC *Dendritic cell, *CIK *Cytokine-induced killer, *AIT *Adoptive immunotherapy, *IFN *Interferon, *OCT *Oral chemotherapy, *TACE *Transcatheter arterial chemoembolization, *PVC *Portal vein chemotherapy, *SOR *Sorafenib, *NA *Non-adjuvant;

When we did network meta-analysis for survival, we used the endpoints including one-year survival, three-year survival and five-year survival. For one-year survival, seventeen studies including 2047 patients were recruited. The network plot showed that most of the studies were compared different treatments with NA, and no loop was presented in different treatments(Fig. 4A). The funnel plot also demonstrated uniform distribution of different comparisons(Fig. 4B). The network analysis showed benefit from TACE [OR, 0.33 (0.14,0.75)] when considering one-year survival. CIK demonstrated the trend of decreasing one-year survival[OR, 2.24 (0.64,7.85)], although no significant difference between CIK and NA. Other treatments including OCT, IRT, IFN, HAIC and ERT didn’t present significant difference between them, although all of them had the trend of increasing one-year survival(Table 4). The ranking of different treatments according to SUCRA was as follows: IFN, 77.0%; IRT 67.8%; TACE, 66.8%; HAIC, 63.1%; ERT, 55.6%; OCT, 38.6%; NA, 24.4%; CIK, 6.7%(Table 5).

**Table 4:** League table of network meta-analysis for one-year survival

TACE	0.43 (0.05,3.96)	0.33 (0.14,0.75)	1.06 (0.22,4.98)	1.87 (0.13,26.21)	0.95 (0.17,5.43)	0.77 (0.09,6.29)	0.15 (0.03,0.66)
2.33 (0.25,21.48)	OCT	0.77 (0.10,6.03)	2.46 (0.21,28.42)	4.37 (0.17,112.29)	2.20 (0.17,29.00)	1.79 (0.11,30.28)	0.34 (0.03,3.82)
3.04 (1.34,6.92)	1.31 (0.17,10.30)	NA	3.21 (0.86,11.97)	5.71 (0.47,69.98)	2.88 (0.62,13.47)	2.34 (0.34,16.17)	0.45 (0.13,1.56)
0.95 (0.20,4.47)	0.41 (0.04,4.70)	0.31 (0.08,1.16)	IRT	1.78 (0.10,30.11)	0.90 (0.12,6.81)	0.73 (0.07,7.52)	0.14 (0.02,0.85)
0.53 (0.04,7.46)	0.23 (0.01,5.89)	0.18 (0.01,2.15)	0.56 (0.03,9.55)	IFN	0.50 (0.03,9.58)	0.41 (0.02,9.72)	0.08 (0.00,1.29)
1.06 (0.18,6.06)	0.45 (0.03,5.97)	0.35 (0.07,1.62)	1.12 (0.15,8.48)	1.98 (0.10,37.58)	HAIC	0.81 (0.07,9.65)	0.15 (0.02,1.13)
1.30 (0.16,10.63)	0.56 (0.03,9.43)	0.43 (0.06,2.95)	1.37 (0.13,14.17)	2.44 (0.10,57.73)	1.23 (0.10,14.61)	ERT	0.19 (0.02,1.90)
6.82 (1.52,30.55)	2.93 (0.26,32.77)	2.24 (0.64,7.85)	7.20 (1.17,44.26)	12.79 (0.78,210.84)	6.46 (0.88,47.15)	5.25 (0.53,52.43)	CIK

*ERT *External radiotherapy, *IRT *Internal radiotherapy, *HAIC *Hepatic artery infusion chemotherapy, *CIK *Cytokine-induced killer, *IFN *Interferon, *OCT *Oral chemotherapy, *TACE *Transcatheter arterial chemoembolization, *NA *Non-adjuvant

**Table 5:** Surface under the cumulative ranking curve (SUCRA) values for one-year survival

Treatment	SUCRA(%)
IFN	77.0
IRT	67.8
TACE	66.8
HAIC	63.1
ERT	55.6
OCT	38.6
NA	24.4
CIK	6.7

*ERT *External radiotherapy, *IRT *Internal radiotherapy, *HAIC *Hepatic artery infusion chemotherapy, *CIK *Cytokine-induced killer, *IFN *Interferon, *OCT *Oral chemotherapy, *TACE *Transcatheter arterial chemoembolization, *NA *Non-adjuvant

**Fig. 5:**
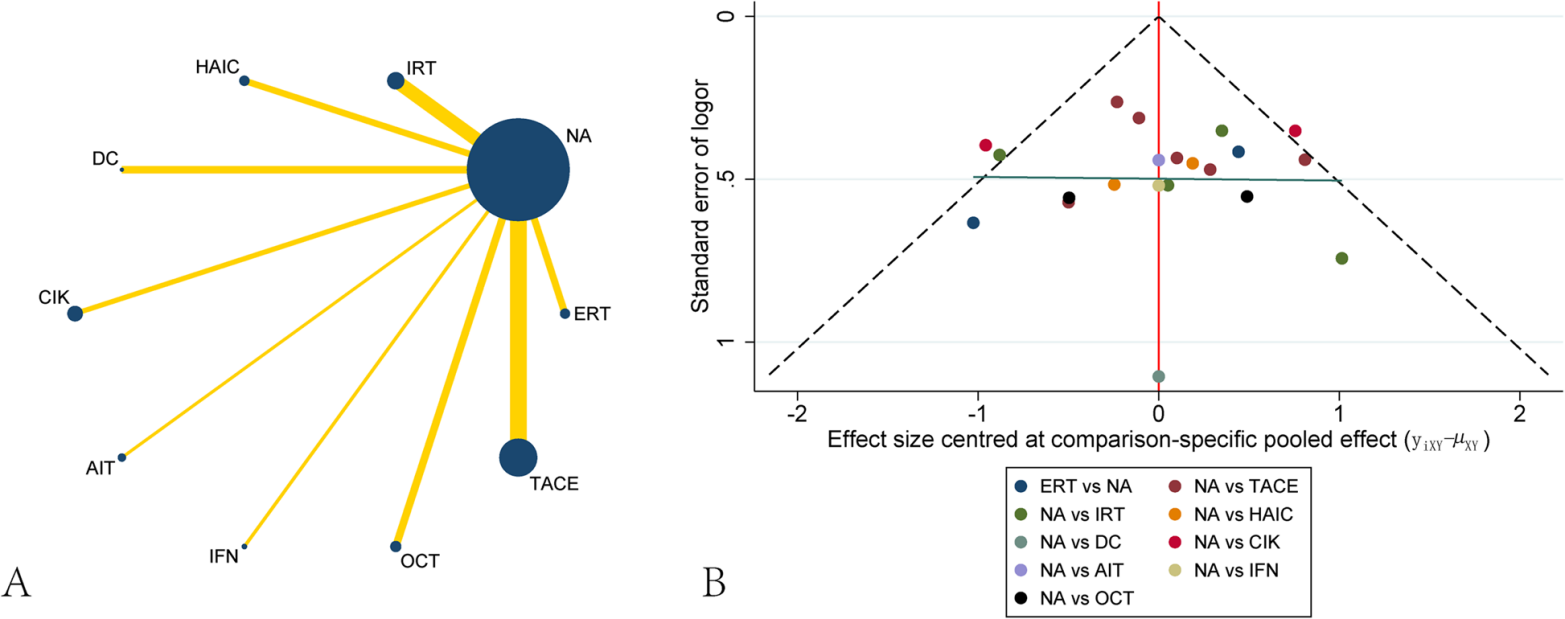
Network plot and funnel plot of studies included in the analysis of three-yea survival. **A** Network plot of studies included in the analysis of three-yea survival. **B** funnel plot of studies included in the analysis of three-yea survival. ERT, external radiotherapy; IRT, internal radiotherapy; HAIC, hepatic artery infusion chemotherapy; DC, dendritic cell; CIK, cytokine-induced killer; AIT, adoptive immunotherapy; IFN, interferon; OCT, oral chemotherapy; TACE, transcatheter arterial chemoembolization; NA, non-adjuvant

For three-year survival, twenty-one studies including 2463 patients were collected. The network plot showed that most of the studies were compared different treatments with NA, and no loop was presented in different treatments(Fig. 5A). The funnel plot also demonstrated uniform distribution of different comparisons(Fig. 5B). The network analysis showed benefit from TACE [OR, 0.51 (0.30,0.86)], IRT[OR, 0.41 (0.20,0.83)] and DC[OR, 0.09 (0.01,0.98)] when considering three-year survival. CIK demonstrated the trend of decreasing three-year survival[OR, 1.59 (0.66,3.84)], although no significant difference between CIK and NA. Other treatments including OCT, IFN, HAIC, ERT and IRT didn’t present significant difference between them, although all of them had the trend of increasing three-year survival(Table 6). The ranking of different treatments according to SUCRA was as follows: DC, 92.3%; IRT, 67.9%; AIT, 66.5%; HAIC, 59.7%; TACE, 57.6%; ERT, 47.0%; IFN, 46.6%; OCT, 35.4%; NA, 19.8%; CIK, 7.1%(Table 7).

**Table 6:** League table of network meta-analysis for three-year survival

TACE	0.65 (0.20,2.10)	0.51 (0.30,0.86)	1.23 (0.51,2.94)	0.82 (0.18,3.76)	1.07 (0.35,3.24)	0.82 (0.26,2.57)	5.64 (0.49,65.33)	0.32 (0.11,0.89)	1.30 (0.31,5.46)
1.54 (0.48,4.99)	OCT	0.78 (0.27,2.23)	1.89 (0.54,6.68)	1.26 (0.21,7.44)	1.64 (0.39,6.91)	1.27 (0.29,5.45)	8.69 (0.64,118.48)	0.49 (0.12,1.94)	2.01 (0.37,10.94)
1.98 (1.17,3.35)	1.28 (0.45,3.67)	NA	2.43 (1.21,4.88)	1.61 (0.38,6.77)	2.11 (0.79,5.61)	1.62 (0.59,4.48)	11.15 (1.02,121.95)	0.63 (0.26,1.53)	2.58 (0.68,9.75)
0.81 (0.34,1.95)	0.53 (0.15,1.87)	0.41 (0.20,0.83)	IRT	0.66 (0.13,3.28)	0.87 (0.26,2.90)	0.67 (0.20,2.28)	4.60 (0.38,55.56)	0.26 (0.08,0.80)	1.06 (0.24,4.78)
1.23 (0.27,5.66)	0.80 (0.13,4.71)	0.62 (0.15,2.61)	1.50 (0.30,7.43)	IFN	1.31 (0.23,7.43)	1.01 (0.17,5.84)	6.92 (0.43,112.56)	0.39 (0.07,2.11)	1.60 (0.23,11.32)
0.94 (0.31,2.85)	0.61 (0.14,2.56)	0.47 (0.18,1.26)	1.15 (0.35,3.84)	0.77 (0.13,4.35)	HAIC	0.77 (0.19,3.16)	5.29 (0.40,70.18)	0.30 (0.08,1.12)	1.22 (0.23,6.38)
1.22 (0.39,3.81)	0.79 (0.18,3.40)	0.62 (0.22,1.70)	1.49 (0.44,5.10)	0.99 (0.17,5.76)	1.30 (0.32,5.32)	ERT	6.87 (0.51,92.31)	0.39 (0.10,1.49)	1.59 (0.30,8.46)
0.18 (0.02,2.05)	0.12 (0.01,1.57)	0.09 (0.01,0.98)	0.22 (0.02,2.63)	0.14 (0.01,2.35)	0.19 (0.01,2.51)	0.15 (0.01,1.96)	DC	0.06 (0.00,0.72)	0.23 (0.01,3.57)
3.14 (1.12,8.78)	2.04 (0.52,8.03)	1.59 (0.66,3.84)	3.85 (1.25,11.89)	2.56 (0.47,13.80)	3.34 (0.89,12.50)	2.58 (0.67,9.91)	17.70 (1.38,226.58)	CIK	4.09 (0.83,20.20)
0.77 (0.18,3.21)	0.50 (0.09,2.71)	0.39 (0.10,1.47)	0.94 (0.21,4.24)	0.63 (0.09,4.43)	0.82 (0.16,4.27)	0.63 (0.12,3.36)	4.33 (0.28,66.86)	0.24 (0.05,1.21)	AIT

*ERT *External radiotherapy, *IRT *Internal radiotherapy, *HAIC *Hepatic artery infusion chemotherapy, *DC *Dendritic cell, *CIK *Cytokine-induced killer, *AIT *Adoptive immunotherapy, *IFN *Interferon, *OCT *Oral chemotherapy, *TACE *Transcatheter arterial chemoembolization, *NA *Non-adjuvant

**Table 7:** Surface under the cumulative ranking curve (SUCRA) values for three-year survival

Treatment	SUCRA(%)
DC	92.3
IRT	67.9
AIT	66.5
HAIC	59.7
TACE	57.6
ERT	47.0
IFN	46.6
OCT	35.4
NA	19.8
CIK	7.1

*ERT *External radiotherapy, *IRT *Internal radiotherapy, *HAIC *Hepatic artery infusion chemotherapy, *DC *Dendritic cell, *CIK *Cytokine-induced killer, *AIT *Adoptive immunotherapy, *IFN *Interferon, *OCT *Oral chemotherapy, *TACE *Transcatheter arterial chemoembolization, *NA *Non-adjuvant

**Fig. 6:**
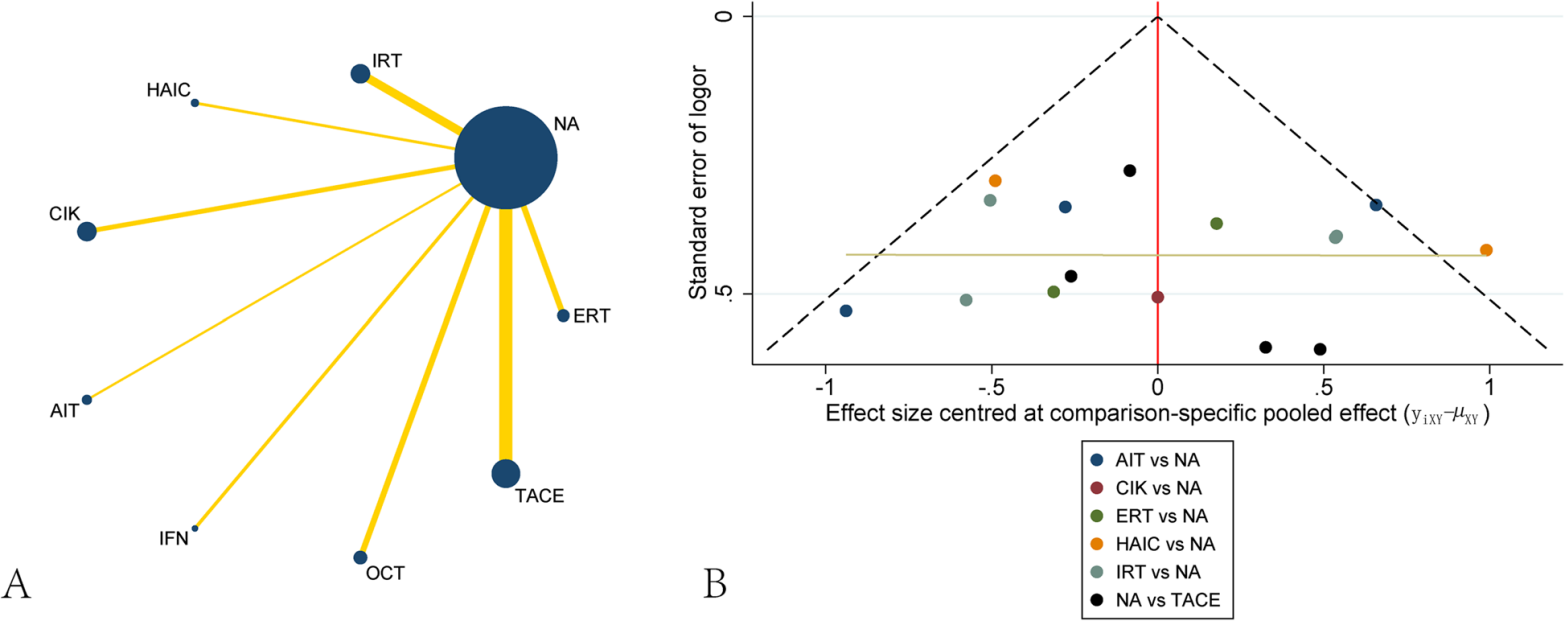
Network plot and funnel plot of studies included in the analysis of five-yea survival. **A** Network plot of studies included in the analysis of five-yea survival. **B** funnel plot of studies included in the analysis of five-yea survival. ERT, external radiotherapy; IRT, internal radiotherapy; HAIC, hepatic artery infusion chemotherapy; CIK, cytokine-induced killer; AIT, adoptive immunotherapy; IFN, interferon; OCT, oral chemotherapy; TACE, transcatheter arterial chemoembolization; NA, non-adjuvant

For five-year survival, Sixteen studies including 1915 patients were collected. The network plot showed that most of the studies were compared different treatments with NA, and no loop was presented in different treatments(Fig. 6A). The funnel plot also demonstrated uniform distribution of different comparisons(Fig. 6B). The network analysis didn’t show any benefit for different treatments when compared to non-adjuvant therapy. TACE[OR, 0.52 (0.25,1.05)], IRT[OR, 0.49(0.23,1.07)], IFN[OR, 0.42(0.10,1.79)], ERT[OR, 0.51(0.19,1.34)] and AIT[OR, 0.77(0.22,2.73)] had the trend of increasing five-year survival, but with no significant difference(Table 6). Other treatments including OCT[OR, 1.01(0.38,2.66)], HAIC[OR, 1.00(0.26,3.77)], and CIK[OR, 1.10(0.44,2.74) didn’t show any benefit(Table 8). The ranking of different treatments according to SUCRA was as follows: IFN, 74.1%; IRT, 71.8%; TACE, 69.5%; ERT, 69.1%; AIT, 47.1%; HAIC, 33.6%; OCT, 31.4%; NA, 27.3%; CIK, 26.1%(Table 9).

**Table 8:** League table of network meta-analysis for five-year survival

TACE	0.51 (0.15,1.71)	0.52 (0.25,1.05)	1.04 (0.37,2.99)	1.24 (0.24,6.32)	0.52 (0.11,2.33)	1.02 (0.31,3.38)	0.47 (0.15,1.49)	0.67 (0.16,2.88)
1.95 (0.59,6.47)	OCT	1.01 (0.38,2.66)	2.03 (0.59,7.05)	2.42 (0.42,14.01)	1.01 (0.19,5.22)	1.98 (0.50,7.80)	0.91 (0.24,3.48)	1.31 (0.27,6.48)
1.93 (0.95,3.94)	0.99 (0.38,2.63)	NA	2.02 (0.93,4.38)	2.41 (0.56,10.37)	1.00 (0.26,3.77)	1.97 (0.75,5.18)	0.91 (0.36,2.25)	1.30 (0.37,4.63)
0.96 (0.33,2.74)	0.49 (0.14,1.70)	0.49 (0.23,1.07)	IRT	1.19 (0.23,6.22)	0.49 (0.11,2.30)	0.98 (0.28,3.37)	0.45 (0.14,1.48)	0.64 (0.15,2.85)
0.80 (0.16,4.08)	0.41 (0.07,2.39)	0.42 (0.10,1.79)	0.84 (0.16,4.39)	IFN	0.42 (0.06,3.00)	0.82 (0.14,4.73)	0.38 (0.07,2.11)	0.54 (0.08,3.75)
1.93 (0.43,8.72)	0.99 (0.19,5.15)	1.00 (0.26,3.77)	2.02 (0.43,9.40)	2.41 (0.33,17.33)	HAIC	1.97 (0.38,10.19)	0.91 (0.18,4.54)	1.30 (0.21,8.17)
0.98 (0.30,3.26)	0.50 (0.13,1.98)	0.51 (0.19,1.34)	1.03 (0.30,3.54)	1.22 (0.21,7.04)	0.51 (0.10,2.62)	ERT	0.46 (0.12,1.74)	0.66 (0.13,3.26)
2.13 (0.67,6.79)	1.10 (0.29,4.19)	1.10 (0.44,2.74)	2.23 (0.67,7.37)	2.65 (0.47,14.84)	1.10 (0.22,5.52)	2.17 (0.57,8.22)	CIK	1.44 (0.30,6.84)
1.48 (0.35,6.35)	0.76 (0.15,3.77)	0.77 (0.22,2.73)	1.55 (0.35,6.85)	1.85 (0.27,12.78)	0.77 (0.12,4.81)	1.51 (0.31,7.45)	0.70 (0.15,3.31)	AIT

*ERT *External radiotherapy, *IRT *Internal radiotherapy, *HAIC *Hepatic artery infusion chemotherapy, *DC *Dendritic cell, *CIK *Cytokine-induced killer, *AIT *Adoptive immunotherapy, *IFN *Interferon, *OCT *Oral chemotherapy, *TACE *Transcatheter arterial chemoembolization, *NA *Non-adjuvant

**Table 9:** Surface under the cumulative ranking curve (SUCRA) values for five-year survival

Treatment	SUCRA
IFN	74.1
IRT	71.8
TACE	69.5
ERT	69.1
AIT	47.1
HAIC	33.6
OCT	31.4
NA	27.3
CIK	26.1

*ERT *External radiotherapy, *IRT *Internal radiotherapy, *HAIC *Hepatic artery infusion chemotherapy, *DC *Dendritic cell, *CIK *Cytokine-induced killer, *AIT *Adoptive immunotherapy, *IFN *Interferon, *OCT *Oral chemotherapy, *TACE *Transcatheter arterial chemoembolization, *NA *Non-adjuvant

## Discussion

Most of the patients with early-stage HCC would undergo curative resection, whereas 50% patients might have disease recurrence within 5 years(Llovet et al. 2016). Studies have identified risk factors that associated with recurrence after curative resection, including macrovascular/microvascular invasion, tumor size greater than 5 centimeters in diameter, multiple nodes, positive resection margin or resection margin less than 1 centimeter, hepatitis B virus infection and AFP greater than 400ng/L(Imamura et al. 2003; Wang et al. 2020; Zeng et al. 2022). Numerous procedures were explored to decrease the recurrence rate and prolong life span. A network meta-analysis included 23 RCTs showed that IRT and HAIC were ranked as the best strategies for preventing recurrence and providing survival benefit(Liu et al. 2021). However, A recent evidence-based management of hepatocellular carcinoma identified 7 RCTs and concluded that adjuvant treatments didn’t improve recurrence-free survival (RFS)(Haber et al. 2021). There needs to be more studies to get more confident conclusions.

In present NMA, we collected thirty-two studies including 5783 patients from a total of 5846 records, which was the largest data for the analysis of adjuvant therapy for post-operative patients with HCC. Furthermore, all the trials included were RCTs with high quality and low bias according to the Cochrane evaluation. When considering the treatments that preventing post-operative recurrence, TACE + PVC[OR, 2.84 (1.15,6.99)] and IRT[OR, 2.63 (1.41,4.91)] were showed to be beneficial for these groups of patients. PVC was considered as an effective strategy in preventing recurrence in patients with portal vein tumor thrombosis(Fan et al. 2005). However, the surgical skill requirement and high risk limited the popularization of the treatment. TACE was confirmed to be disease-free survival benefit in MVI-positive patients, but weekly supports in patients without portal venous tumor thrombus(PVTT) in meta-analysis(Huo et al. 2020; Shen et al. 2020; Yang et al. 2021). Alpha fetoprotein level, systemic inflammation response index, alanine aminotransferase, tumour diameter and portal vein tumour thrombus were also confirmed to be independent prognostic factors of HCC early recurrence in patients with MVI who underwent TACE(Mao et al. 2022). However, PVC promoted median time to recurrence and OS in HCC patients with PVTT(Gao et al. 2019). The combination of TACE and PVC provided favorable recurrence free in our study, which might be related with the mixed baseline in studies recruited, and TACE plus PVC would decrease the recurrence rate in all patients with HCC after curative resection. The IRT included I^131^-mAb, I^131^-lipiodol and I^125^ seeds, obviously three strong RCTs provided the benefits for all the internal radiotherapy(Chen et al. 2013; Lau et al. 1999; Li et al. 2020a). However, some studies didn’t show any recurrence-free or OS benefit(Chung et al. 2013; Furtado et al. 2015).The implantation of internal radioactive source is also technique requirement which might be restriction of the application. The ranking of different treatments according to SUCRA was as follows: TACE + PVC, 88.0%; IRT, 82.3%; ERT, 72.8%; AIT, 71.8%; Huaier, 59.4%; HAIC, 58.7%; TACE, 51.0%, IFN, 48.0%; OCT, 39.0%; CIK, 36.7%; DC, 30.7%; BCAA, 30.5%; NA, 23.4%; SOR, 7.8%. From this data, we found out that adjuvant sorafenib was not a good choice for the patients to prevent recurrence according to the STORM trial(Bruix et al. 2015). Apart from the treatments collected in this study, immune checkpoint inhibitors(Kudo et al. 2022), antiviral therapy(Yin et al. 2013), Lenvatinib(Bai et al. 2022), and traditional herbal medicine(Zhai et al. 2018) also indicated recurrence free or OS benefit in single arm or prospective studies.

As for comparison of one-year survival, three-year survival and five-year survival in different treatments, TACE was considered to be favorable prognosis for one-year survival [OR, 0.33 (0.14,0.75)] and three-year survival[OR, 0.51 (0.30,0.86)], which was also confirmed by the meta-analysis before(Huo et al. 2020; Shen et al. 2020; Yang et al. 2021). IRT was considered as the good choice when evaluating three-year survival[OR, 0.41 (0.20,0.83)]. As for DC in the adjuvant therapy, the small size of the trial which might be selection bias for this result and more confident evidence needs to be performed(Matsui et al. 2021). The primary outcome of studies included in this analysis is the prevention of recurrence. However, the second outcomes in different studies are diverse. For example, the comparison of one-year survival just includes IFN, IRT, TACE, HAIC, ERT, OCT and CIK, which is fewer than the primary outcome. Moreover, the basic characteristics in IFN groups are better than other comparison, which might cause the difference and bias.

However, there are limitations in this NMA. Firstly, the basic characteristic for each trial is not paired enough. For example, some trials include patients with vascular invasive, surgical margins are diverse for different trials. Microvascular invasion and/or positive/narrow surgical margins are independent risk factors for HCC patients under hepatic resection(Hwang et al. 2023; Liu et al. 2023; Wang et al. 2023). The different patients included in trials might cause the bias of the comparison. For example, most of the patients recruited in ERT are positive surgical margins or Margin ≤ 1 cm, and the treatment of ERT might bring significant benefit for these patients(Shi et al. 2022; Yu et al. 2014). The benefit for IRT is the same as ERT(Chen et al. 2013; Chung et al. 2013; Li et al. 2020a). Most of the patients recruited in HAIC are MVI positive, however, nearly none of the patients collected in CIK are MVI negative, which might cause the significant different effect when comparing with observation(Li et al. 2020b; Xu et al. 2016). Apart from the diverse basic characteristic between different comparison, the patients included in the same comparison are different(Hirokawa et al. 2020; Li et al. 2020b). Secondly, the number of patients collected in each group are enormous diversity, which might bring bias when analyzing the data. Thirdly, some treatment such as IRT including different internal radioactive source which maybe different effectiveness. Furthermore, some treatment is single trial with limited number of patients recruited, which might restrict the application of the treatment.

In conclusion, the effectiveness for different adjuvant treatments in post-operative patients with HCC varies in trials. However, the adjuvant treatments have the trend of preventing recurrence and increase overall survival, although most of the procedures are no significant difference when compared to non-adjuvant therapy. This NMA found that TACE + PVC and IRT were considered as the best way to decrease recurrence rate. TACE, IRT and DC were preferred when considering the extend for life span. There needs to be more large scale of studies to confirm the results.

## Data Availability

The original contributions presented in the study are included in the article, further inquiries can be. directed to the corresponding author.
